# Arterial dP/dt_max _accurately reflects left ventricular contractility during shock when adequate vascular filling is achieved

**DOI:** 10.1186/1471-2261-12-13

**Published:** 2012-03-01

**Authors:** Philippe Morimont, Bernard Lambermont, Thomas Desaive, Nathalie Janssen, Geoffrey Chase, Vincent D'Orio

**Affiliations:** 1 Medical Intensive Care Unit, University Hospital of Liège, Liège, Belgium; 2 Faculty of Sciences, University of Liège, Liège, Belgium; 3 Emergency Department, University Hospital of Liège, Liège, Belgium; 4 Mechanical Engineering Department, University of Canterbury, Christchurch, New Zealand

**Keywords:** Left ventricular function, Aortic pressure, Septic cardiomyopathy, Preload responsiveness, Endotoxin-induced shock

## Abstract

**Background:**

Peak first derivative of femoral artery pressure (arterial dP/dt_max_) derived from fluid-filled catheter remains questionable to assess left ventricular (LV) contractility during shock. The aim of this study was to test if arterial dP/dt_max _is reliable for assessing LV contractility during various hemodynamic conditions such as endotoxin-induced shock and catecholamine infusion.

**Methods:**

Ventricular pressure-volume data obtained with a conductance catheter and invasive arterial pressure obtained with a fluid-filled catheter were continuously recorded in 6 anaesthetized and mechanically ventilated pigs. After a stabilization period, endotoxin was infused to induce shock. Catecholamines were transiently administrated during shock. Arterial dP/dt_max _was compared to end-systolic elastance (Ees), the gold standard method for assessing LV contractility.

**Results:**

Endotoxin-induced shock and catecholamine infusion lead to significant variations in LV contractility. Overall, significant correlation (r = 0.51; *p *< 0.001) but low agreement between the two methods were observed. However, a far better correlation with a good agreement were observed when positive-pressure ventilation induced an arterial pulse pressure variation (PPV) ≤ 11% (r = 0.77; *p *< 0.001).

**Conclusion:**

While arterial dP/dt_max _and Ees were significantly correlated during various hemodynamic conditions, arterial dP/dt_max _was more accurate for assessing LV contractility when adequate vascular filling, defined as PPV ≤ 11%, was achieved.

## Background

Sepsis-induced myocardial dysfunction occurs more frequently than expected while its severity is often underestimated [1]. Indeed, quantification of left ventricular (LV) inotropic function during sepsis is an ongoing preoccupation of clinicians. The theoretical gold standard for assessment of LV contractility is the end-systolic pressure volume relation (ESPVR). The slope of the relation defines the maximum elastance, also called end-systolic elastance (Ees), a load-independent index of LV contractility [2]. However, this method is difficult to apply in clinical practice because requiring preload reduction and catheterization of the left ventricle with a high-fidelity pressure catheter. Several other contractility indices have been proposed but most of them are influenced by cardiac loading conditions [3-8]. A relative exception to this is the peak first derivative of LV pressure (LV dP/dt_max_) which is relatively afterload independent [9]. Most critically ill patients with hemodynamic instability are instrumented with a femoral fluid-filled catheter for accurate arterial pressure monitoring. dP/dt_max _can be derived from the arterial pressure curve (the maximal ascending slope of the peripheral arterial pressure curve). However, the use of arterial dP/dt_max _as an index of LV contractility remains questionable because dP/dt_max _is derived from arterial pressure obtained with a fluid-filled catheter and is influenced by preload and vascular filling conditions [4,10]. Vascular filling and fluid requirement in critically ill patients are usually assessed by positive-pressure ventilation-induced arterial pulse pressure variation (PPV), which is a sensitive and specific predictor of preload responsiveness. PPV is measured over a single respiratory cycle and defined as the maximal pulse pressure (systolic-diastolic pressure) less the minimal pulse pressure divided by the average of these two pressures. In hypovolemic states, PPV due to cycling pressure gradient from mechanical ventilation is high. However, when adequate vascular filling is obtained, PPV is low [11]. The purpose of this study was to investigate whether arterial dP/dt_max _derived from fluid-filled femoral artery catheter can be used to assess LV contractility in different hemodynamic conditions. To test this hypothesis, arterial dP/dt_max_, LV dP/dt_max _and Ees were compared during various alterations in LV contractile function resulting from endotoxin-induced shock and catecholamine infusion. PPV was continuously monitored to assess the vascular filling.

## Methods

All experimental procedures and protocols used in this investigation were reviewed and approved by the ethical committee of the Medical Faculty of the University of Liege and conformed to the Guide for the Care and Use of Laboratory Animals published by the US National Institutes of Health (NIH Publication No. 85-23, revised 1996).

### Experimental model

Experiments were performed on 6 healthy pure Pietran pigs of either sex weighing from 16 to 28 kg. The animals were premedicated with intramuscular administration of tiletamine (250 mg) and zolazepam (250 mg). Anaesthesia was then induced and maintained by a continuous infusion of sufentanil (0.5 μg/kg/h) and pentobarbital (5 mg/kg/h). Spontaneous movements were prevented by cisatracurium (0.1 mg/kg/h). After endotracheal intubation via a cervical tracheostomy, the pigs were connected to a volume-cycled ventilator (Datex Ohmeda, Engström Carestation, General Electric, USA) set to deliver a tidal volume of 10 ml/kg at a respiratory rate of 20/min with a FiO_2 _of 0.4 and a PEEP of 5 cm H2O. A 7F, multi-electrode (9-mm interelectrode distance) conductance micromanometer-tipped catheter (Scisense, Canada) was inserted through the left carotid artery into the left ventricle and positioned so that all electrodes remained in the LV cavity. A central venous line was inserted into the right jugular vein and placed inside the superior vena cava. Arterial blood pressure was monitored via a 4F fluid-filled catheter (Pulsiocath, Pulsion Medical System, Germany) inserted into the right femoral artery. A 6F Fogarty balloon catheter (Baxter Healthcare Corp., Oakland, CA, USA) was advanced into the inferior vena cava through a right femoral venotomy. Inflation of this balloon produced a gradual preload reduction.

### Experimental protocol

After surgical preparation, the animals were allowed to stabilize for 30 min ('basal' period). Hemodynamic data including mean arterial blood pressure, heart rate (HR), cardiac output (CO), LV volume and pressure, were continuously recorded. Then, the animals had a 0.5 mg/kg intravenous infusion of a freshly prepared endotoxin solution (lipopolysaccharide from E.coli serotype 0127:B8, Sigma, St Louis, MO, USA) over 30 min ('endo' period). When systolic arterial pressure significantly dropped, dobutamine (5 mcg/kg/min) and norepinephrine (0.05 mcg/kg/min) were administrated during 60 minutes ('catechol' period) and then stopped ('shock' period). Fluid administration with Hartmann's solution was continuously controlled by preload responsiveness. When PPV was ≤ 11%, animals were considered as adequately filled.

### Data collection and analysis

All analog signals were continuously digitalized and recorded (Notocord-hem evolution, Notocord, Paris, France). During each period of measurement, three transient occlusions of the inferior vena cava using the Fogarty balloon were performed during apnea. Analysis of the signals was performed offline. Arterial dP/dt_max _and LV_dP/dt_max _were calculated on 6 steady-state cycles just before occlusion of the vena cava. These indices were compared with the gold-standard Ees, calculated during transient preload reduction.

### Statistical analysis

Arterial dP/dt_max_, LV dP/dt_max _and Ees were compared using linear regressions. A normalized Bland-Altman test (Statistica version 7, StatSoft) was also performed. Changes in hemodynamic parameters were evaluated by a repeated-measures analysis of variance. Data were expressed as mean ± standard deviation (SD).

## Results

**Figure 1:**
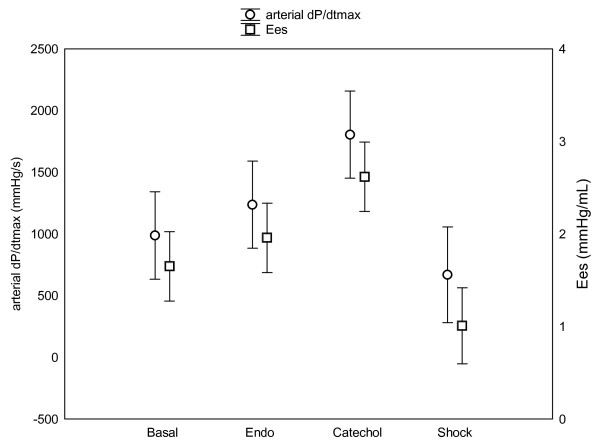
**LV contractility assessed by both Ees and arterial dP/dt_max_**. Basal conditions ('basal'), immediately after endotoxin infusion ('endo'), during shock with and without catecholamine infusion ('catechol' and 'shock' respectively). Values are given as mean ± SD. All directional changes in contractility were significant (*p *< 0.05) for each challenge, except between 'basal' and 'endo'.

The effects of endotoxin infusion and catecholamine administration on arterial pressure, HR, ejection fraction (EF) and cardiac output (CO) are summarized in Table 1. The evolution of LV contractility assessed by both Ees and arterial dP/dt_max _is shown in Figure 1. Ees significantly decreased from 1.63 ± 0.4 to 1.18 ± 0.55 mm Hg/mL during the state of shock. Arterial and LV dP/dt_max _significantly decreased from 1004 ± 41 and 2414 ± 514 to 795 ± 305 and 1235 ± 224 mm Hg/sec, respectively. However, during catecholamine infusion, Ees significantly increased to 2.5 ± 0.77 mm Hg/mL and arterial and LV dP/dt_max _significantly increased to 1872 ± 491 and 3181 ± 485 mm Hg/sec, respectively.

**Table 1:** Hemodynamic data

	SAP (mm Hg)	DAP (mm Hg)	HR (b/min)	EF (%)	CO (L/min)
Basal	113 ± 12	69 ± 9	98 ± 12	55 ± 7	4.7 ± 0.9

Endo	109 ± 9	64 ± 11	106 ± 16	57 ± 8	4.9 ± 0.8

Catechol	88 ± 12 *	41 ± 8 *	129 ± 13	69 ± 7 *	6.3 ± 0.9 *

Shock	46 ± 15 *	24 ± 10 *	77 ± 16	41 ± 7	2.0 ± 1.1 *

SAP = systolic arterial pressure, DAP = diastolic arterial pressure, HR = heart rate, EF = left ventricular ejection fraction, CO = cardiac output, *P < 0.01 compared to basal

**Figure 2:**
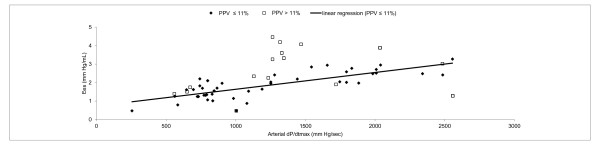
**Linear regression between arterial dP/dt_max _and Ees**.

**Figure 3:**
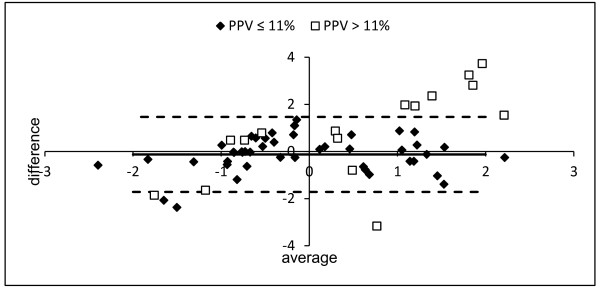
**Normalized Bland-Altman plot of the agreement between Ees and arterial dP/dt_max_**. Average = (Ees° + arterial dP/dt_max_°)/2 and difference = Ees° - arterial dP/dt_max_° where X° is the normalized value of X [X° = (value of X - mean of X)/standard deviation of X]. Lines represent mean difference (solid lines) and 95% confidence interval (light dashed line) (PPV ≤ 11%).

**Figure 4:**
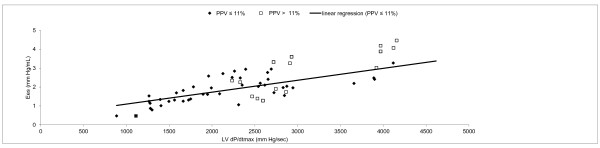
**Linear regression between left ventricular (LV) dP/dt_max _and Ees**.

Overall, arterial dP/dt_max _and Ees were significantly correlated (r = 0.51, *p *< 0.001) but there was low agreement (Figures 2 and 3). LV dP/dt_max _and arterial dP/dt_max _were significantly correlated (r = 0.58, *p *< 0.001) but arterial dP/dt_max _systematically underestimated LV dP/dt_max_. (bias = 1018 ± 364 mmHg/sec). LV dP/dt_max _and Ees were significantly correlated (r = 0.78, *p *< 0.001) (Figure 4).

**Figure 5:**
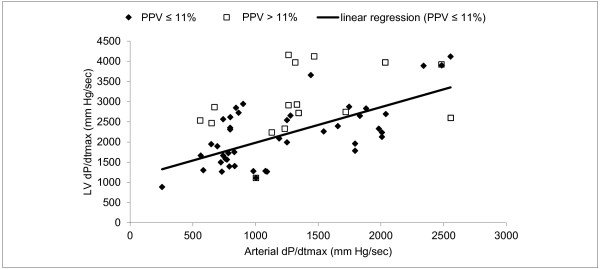
**Linear regression between left ventricular (LV) dP/dt_max _and arterial dP/dt_max_**.

When adequate filling (PPV ≤ 11%) was obtained, a far better correlation between arterial dP/dt_max _and Ees was found (r = 0.77, *p *< 0.001) (Figure 2). In that case, normalized Bland-Altman analysis revealed a good agreement between the two methods (Figure 3). Correlation between LV dP/dt_max _and arterial dP/dt_max _was also improved when adequate filling was achieved (r = 0.66, *p *< 0.001) while correlation between LV dP/dt_max _and Ees did not significantly changed (r = 0.76, *p *< 0.001) (Figure 5).

## Discussion

Determination of LV contractility is a cornerstone in clinical practice [6,12]. Numerous methods for assessing LV contractility have been reported but none have been adequately validated in clinical practice, or require the presence of an intraventricular pressure catheter, prohibiting routine use in clinical practice [13,14]. The gold standard method, Ees, requires both ventricular pressure and volume measurements on a beat to beat basis with preload variation [2]. Single beat analysis has been developed, but requires the whole ventricular pressure waveform [15]. dP/dt_max _can be easily calculated in clinical practice but is sensitive to both LV contractility and preload [9]. In the present study, we tested whether arterial dP/dt_max_, derived from femoral fluid-filled catheter, was accurate for assessing LV contractility. While LV dP/dt_max _is considered as a good index of LV contractility despite its preload dependence, little is known about arterial dP/dt_max _[16,17]. One study performed in perioperative patients found that arterial dP/dt_max _and LV dP/dt_max _were significantly correlated and concluded that changes in arterial dP/dt_max _were accurate for assessing changes LV contractility [18]. To the best of our knowledge, despite its wide use in critically ill patients, arterial dP/dt_max _has never been directly compared with Ees during changes in LV function, at different levels of vascular filling. Our results demonstrated that there was significant correlation between arterial dP/dt_max _and Ees. Furthermore, a far better correlation with a good agreement between arterial dP/dt_max _and Ees were observed when adequate vascular filling was achieved. Similar improvement was observed between arterial and LV dP/dt_max_. However, correlation between LV dP/dt_max _and Ees did not significantly change when adequate vascular filling was achieved. Arterial dP/dt_max _is an ejection phase index depending on arterial compliance and waves reflections from periphery to aorta. All factors that may affect arterial compliance and waves reflections (vascular filling conditions, vasoactive agents) may also affect arterial dP/dt_max _independently of LV contractile function. As a result, the combination of fluid responsiveness and changes in arterial compliance and waves reflections due to endotoxin and/or catecholamines could enhance discrepancies between arterial and LV dP/dt_max _and consequently between arterial dP/dt_max _and the reference method, Ees [19]. In this study, fluid administration was directed by PPV.

Adequate vascular filling was defined as PPV ≤ 11%. On the basis of clinical settings, this PPV threshold value allows the best discrimination between responders and nonresponders to intravascular fluid administration [20-23]. In perioperative patients, De Hert et al. showed that changes in femoral dP/dt_max _accurately reflected changes in LV dP/dt_max _during various interventions. However, absolute values of LV contractility are required for potential ventriculo-arterial interaction analysis [24]. These authors also found that leg elevation induced significant increase in central venous pressure and LV end-diastolic pressure, but arterial and LV dP/dt_max _remained unaltered [18]. However, it is well recognized that static indices (like central venous pressure or LV end-diastolic pressure) are poor indicators of vascular filling and preload responsiveness [11]. Masutani et al. showed that LV dP/dt_max _can be predicted from aortic dP/dt_max _but their method requires aortic impedance which is difficult to calculate in clinical practice [25]. Therefore, assessing LV contractility from arterial dP/dt_max_, when adequate vascular filling is achieved, could be a simple and accurate method with the potential for ventriculo-arterial interaction analysis.

Other methodological issues should be taken into account. First, the use of a fluid-filled catheter could be another source of discrepancy between arterial dP/dt_max _and Ees. As pointed out by numerous authors, pressure waves measured with a fluid-filled catheter have to be interpreted cautiously, because the pressure waveform may be distorted by the dynamic response of the catheter. By taking care of a properly flushed catheter system and by filtering out artifacts, the catheter response were optimized in our study [26]. Secondly, arterial and LV dP/dt_max _could also be influenced by heart rate. Heart rate variability was not significant enough to analyze its influence on arterial dP/dt_max _in the present observations.

## Conclusions

Arterial dP/dt_max_, the minimally invasive method derived from femoral artery fluid-filled pressure catheter and Ees, the reference method for assessing LV contractility, derived from intraventricular conductance micromanometer-tipped catheter, were significantly correlated over a wide range of hemodynamic conditions resulting from endotoxin-induced shock and catecholamine infusion. However, arterial dP/dt_max _was more accurate for assessing LV contractility when adequate vascular filling, defined as PPV ≤ 11%, was achieved. Using dynamic indices to ensure adequate vascular filling, LV contractility could be accurately predicted by arterial dP/dt_max _derived from femoral artery fluid-filled pressure catheter in critically ill patients.

## Authors' contributions

PM conceived the study, participated in the experiments, analyzed the data and drafted the manuscript. BL and TD participated in the experiments, analyzed the data and helped to draft the manuscript. NJ participated in the experiments. GC participated in the design of the study and helped to draft the manuscript. VD participated in the design and coordination of the study and helped to draft the manuscript. All authors read and approved the final version of the manuscript.

## Pre-publication history

The pre-publication history for this paper can be accessed here:

http://www.biomedcentral.com/1471-2261/12/13/prepub
